# The influence of *CYP3A*, *PPARA*, and *POR* genetic variants on the pharmacokinetics of tacrolimus and cyclosporine in renal transplant recipients

**DOI:** 10.1007/s00228-014-1656-3

**Published:** 2014-03-22

**Authors:** Ingrid Lunde, Sara Bremer, Karsten Midtvedt, Beata Mohebi, Miriam Dahl, Stein Bergan, Anders Åsberg, Hege Christensen

**Affiliations:** 1 Department of Pharmaceutical Biosciences, School of Pharmacy, University of Oslo, Box 1068, Blindern, 0316 Oslo, Norway; 2 Department of Medical Biochemistry, Oslo University Hospital, Rikshospitalet, Oslo, Norway; 3 Department of Transplant Medicine, Oslo University Hospital, Rikshospitalet, Oslo, Norway; 4 Department of Pharmacology, Oslo University Hospital, Rikshospitalet, Oslo, Norway

**Keywords:** Calcineurin inhibitors, Pharmacokinetics, *CYP3A*, *POR*, *PPARA*, Kidney recipients

## Abstract

**Purpose:**

Tacrolimus (Tac) and cyclosporine (CsA) are mainly metabolized by CYP3A4 and CYP3A5. Several studies have demonstrated an association between the *CYP3A5* genotype and Tac dose requirements. Recently, *CYP3A4, PPARA*, and *POR* gene variants have been shown to influence CYP3A metabolism. The present study investigated potential associations between CYP3A5*3, CYP3A4*22, PPARA c.209-1003G>A and c.208 + 3819A>G, and POR*28 alleles and dose-adjusted concentrations (C/D) of Tac and CsA in 177 renal transplant patients early post-transplant.

**Methods:**

All patients (*n* = 177) were genotyped for CYP3A4*22, CYP3A5*3, POR*28, *PPARA* c.209-1003G>A, and *PPARA* c.208 + 3819A>G using real-time polymerase chain reaction (PCR) and melting curve analysis with allele-specific hybridization probes or PCR restriction fragment length polymorphisms (RFLP) methods. Drug concentrations and administered doses were retrospectively collected from patient charts at Oslo University Hospital, Rikshospitalet, Norway. One steady-state concentration was collected for each patient.

**Results:**

We confirmed a significant impact of the CYP3A5*3 allele on Tac exposure. Patients with POR*28 and *PPARA* variant alleles demonstrated 15 % lower (*P* = 0.04) and 19 % higher (*P* = 0.01) Tac C_0_/D respectively. CsA C_2_/D was 53 % higher among CYP3A4*22 carriers (*P* = 0.03).

**Conclusion:**

The results support the use of pre-transplant *CYP3A5* genotyping to improve initial dosing of Tac, and suggest that Tac dosing may be further individualized by additional *POR* and *PPARA* genotyping. Furthermore, initial CsA dosing may be improved by pre-transplant CYP3A4*22 determination.

**Electronic supplementary material:**

The online version of this article (doi:10.1007/s00228-014-1656-3) contains supplementary material, which is available to authorized users

## Introduction

Calcineurin inhibitors (CNI), cyclosporine (CsA) and tacrolimus (Tac), are potent immunosuppressive drugs and are widely used in solid organ transplant recipients [1]. Both drugs are characterized by a narrow therapeutic window and high interindividual pharmacokinetic variability [2]. Consequently, therapeutic drug monitoring (TDM) is mandatory to optimize CNI therapy in transplant recipients. However, patients still experience significant CNI over- or underexposure in the critical immediate phase after transplantation.

Part of the great variability in CNI pharmacokinetics among individuals may be explained by differences in genes encoding drug metabolizing enzymes or drug transporters [3]. Both CsA and Tac are metabolized by cytochrome P450 3A (CYP3A) enzymes. The CYP3A5 activity is largely determined by the single nucleotide variant (SNV) CYP3A5*3 (c.219-237A>G; rs776746), which results in alternate mRNA splicing and a truncated and non-functional protein [4, 5]. The CYP3A5*3 variant is the predominant allele in many populations, and the majority of Caucasians (approximately 80 %) lack functional CYP3A5 [4–6]. The association between *CYP3A5* genotype and CNI pharmacokinetics is well established [7–11], and patients expressing functional CYP3A5 (one or two CYP3A5*1 alleles), need approximately double starting doses of Tac [12]. CsA appears to be oxidized predominantly by CYP3A4 [13]. However, some of the major CsA metabolites are also formed by CYP3A5, and the *CYP3A5* genotype has been shown to have a significant impact on CsA pharmacokinetics [7, 8, 12]. The expression and activity of the CYP3A4 enzyme varies widely among individuals, but the contribution of specific genetic factors remains uncertain. A recent study identified a functional SNV in intron 6 of the *CYP3A4* gene (c.522-191C>T; rs35599367; CYP3A4*22) associated with reduced CYP3A4 activity [14, 15]. The allele frequency is relatively low in Caucasians (3–8 %), but nonetheless clinically relevant in patients carrying the CYP3A4*22 allele [16–19].

Genes located outside the *CYP3A* locus may also influence CYP3A phenotype. Two sequence variants in the gene encoding the nuclear receptor peroxisome proliferator-activated receptor alpha (PPAR-alpha) have recently been recognized as potential contributors to intra- and inter-individual variability in CYP3A expression and activity [14, 16]. The *PPARA* variants, c.209-1003G>A (rs4253728) and c.208 + 3819A>G (rs4823613), have been reported to explain 8–9 % of the variability in hepatic CYP3A activity in humans [16].

Cytochrome P450 oxidoreductase (POR) is another system influencing CYP3A activity. POR is a microsomal electron transfer flavoprotein and an indispensable element of a variety of CYP enzymes, and other enzymatic complexes [20]. Human *POR* is highly polymorphic (http://www.cypalleles.ki.se/por.htm) [21] and the most common sequence variant, POR*28 (c.1508C>T; rs1057868), induces an amino acid substitution (p.Ala503Val), which influences the electron binding moiety of POR [22]. POR*28 has been associated with different effects depending on the CYP enzyme and substrate investigated [23–26]. CYP3A5 expressers carrying one or two POR*28 alleles have shown a 45 % lower midazolam metabolic ratio [23] and higher Tac dose requirements compared with CYP3A5 expressers without POR*28 [24].

The aim of the present study was to assess the effect of the CYP3A5*3, CYP3A4*22, PPARA c.209-1003G>A, *PPARA* c.208 + 3819A>G, and POR*28 alleles on Tac and CsA dose-adjusted concentrations (C/D) in renal transplant recipients early post-transplant.

## Materials and methods

### Patients

The patients received immunosuppressive treatment based on either CsA or Tac, in combination with mycophenolate and steroids. None was concomitantly treated with potential CYP3A4 inhibiting drugs or statins, but all received proton pump inhibitors at the time of drug concentration measurement. TDM was performed at least twice weekly in this early post-transplant phase and Tac and CsA doses were individually adjusted to achieve predefined target ranges; Tac trough concentrations between 3 and 7 μg/L and CsA C_2_ concentrations between 800 and 1,100 μg/L respectively.

At our transplant center all patients are scheduled for a routine in-depth examination at the research laboratory at 8 weeks and 1 year post-transplantation. From 2 January to 2 July 2012 a total of 229 patients met for an 8-week or 1-year examination. Two hundred patients gave written informed consent prior to inclusion. Of these 200 patients only 42 had CsA trough concentrations measured in the relevant post-transplant period and were not included in this analysis. Adequate data from 158 patients (Tac, *n* = 123 /CsA, *n* = 35) were used in the present analysis in addition to data from 19 CsA patients previously presented (NCT00139009) [27].

The study was approved by the regional ethics committee and performed in accordance with local laws and regulations.

### Study design

Drug concentrations and administered doses were retrospectively collected from patient charts at Oslo University Hospital, Rikshospitalet, Norway. One steady-state concentration was collected for each patient in the early post-transplant phase, i.e. 2 to 7 weeks after transplantation. Steady-state was defined as at least 3 days after last dose adjustment for Tac and 4 days for CsA.

All patients (*n* = 177) were genotyped for the sequence variants CYP3A5*3, CYP3A4*22, *PPARA* c.209-1003G>A, *PPARA* c.208 + 3819A>G, and POR*28.

### Analytical methods

Whole blood Tac concentrations were measured using the CMIA (chemiluminescent microparticle immunoassay) on the Architect instrument (Abbott Laboratories, Lake Forest, IL, USA) and CsA concentrations using the CEDIA PLUS assay (Cloned Enzyme Donor Immunoassay; Microgenics Corporation, Fremont, CA, USA) on a Modular P800 analyzer (Roche Diagnostics, Rotkreuz, Switzerland).

### Genotype analyses

Genomic DNA was extracted from whole blood samples using the MagNA Pure LC DNA Isolation Kit I (Roche) on the automated MagNA Pure LC Instrument (Roche). Genotyping of POR*28, *PPARA* c.209-1003G>A, and *PPARA* c.208 + 3819A>G were performed using polymerase chain reaction restriction fragment length polymorphism (PCR-RFLP) methods. Primer sequences and restriction enzymes are listed in Supplementary Material 1. PCR was performed using DNA Engine Dyad® Thermal Cycler (Bio-Rad Laboratories, Hercules, CA, USA). PCR products were digested with 1 U of the associated restriction enzyme (Supplementary Material 1), and the digested products were separated by electrophoresis on a 3 % agarose gel and visualized under ultraviolet light after staining with GelRed™. The assays were validated by sequencing a selection of wild-type and variant samples. CYP3A5*3 (NM_000777.3:c.219-237A>G) and CYP3A4*22 (NM_001202855.2:c.522-191C>T) alleles were analyzed using real-time PCR and melting curve analysis with allele-specific hybridization probes on the LightCycler® 480 instrument (Roche) as previously described for CYP3A5*3 [28]. Amplification conditions, oligonucleotide sequences, and reaction mixtures are listed in Supplementary Materials 2, 3, and 4. Absence of variant alleles was interpreted as the presence of the wild-type allele (*1).

### Data and statistical analyses

The potential association between CYP3A5*3, CYP3A4*22, PPARA, POR*28 genotypes and steady state dose-adjusted Tac C_0_ (C_0_/D, μg*L^-1^/mg) or CsA C_2_ (C_2_/D, μg*L^-1^/mg) concentration, 2–7 weeks post-transplantation was investigated. Dose-adjusted concentrations were calculated by dividing the C_0_ or C_2_ by the evening or morning dose respectively.

Statistical analyses were performed using SPSS software (version 20, IBM SPSS Statistics, Chicago, IL, USA). The Kolmogorov–Smirnov test was used to evaluate the distribution of continuous data, and if appropriate, data were logarithmically transformed to obtain normal distribution. The impact of CYP3A5*3, CYP3A4*22, *PPARA* c.209-1003G>A, *PPARA* c.208 + 3819A>G, and POR*28 alleles on dose-adjusted Tac or CsA concentrations was investigated by a univariate analysis of variance (ANOVA). ANOVA coefficients were back-transformed to present geometric means and SEM. The interaction effect between the different sequence variants was evaluated and excluded from the analysis if not statistically significant. Associations between categorical data (e.g., *PPARA* c.208 + 3819A>G genotype) were analyzed using Fisher’s exact test. Spearman’s rho was assessed to study the correlation between continuous and dichotomous variables. *P* values less than 0.05 were considered to be statistically significant.

## Results

### Patients

Data from the 177 (Tac, *n* = 123/CsA, *n *= 54) included patients were obtained on average 18±5 days after transplantation. Patient demographics for the two groups are summarized in Table 1. The patients included were not demographically different from those 79 who during the same period underwent the in-depth evaluation, but were not included (data not shown). Time after transplantation, age, weight, height, body mass index (BMI), bilirubin levels, diabetes mellitus (DM) status or use of dihydropyridine derivatives did not significantly correlate with drug concentration, and these covariates were therefore not included in the multivariate analyses.

**Table 1:** Demographic data, median (range), at the time of data collection

Demographics	Tac C_0_ group *n* = 123	CsA C_2_ group *n* = 54
Male/female (*n*)	87/36	41/13
Age, years	48 (20–79)	60 (21*–*81)
Height (m)	1.75 (1.54–2.06)	1.76 (1.55*–*1.90)
Weight (kg)	74.2 (43.6–158.0)	76.7 (46.9*–*118.0)
BMI (kg/m^2^)	24.7 (16.4–40.7)	24.4 (19.5*–*38.9)
Diabetes mellitus (*n*)	21	5
Bilirubin (μmol/L)	6 (2–15)	10 (5*–*25)
Treated with dihydropyridine derivatives (*n*)	70/123	22/54
Treated with statins (*n*)	0/123	0/54
Treated with glucocorticoids (*n*)	123/123	54/54
Treated with proton pump inhibitor (*n*)	123/123	54/54
CNI dose (mg/day)	3.5 (1.5–9.0)	175 (100*–*425)
Blood concentration (μg/L)	7.0 (4.1–13.8)	1252 (620*–*3,240)
C/D ratio^a^ (μg*L^-1^/mg)	2.0 (0.6–5.5)	7.2 (2.2*–*16.2)
Time after transplantation (days)	15 (14–31)	18.5 (14*–*48)

*BMI* body mass index (kg/m^2^), *CNI* calcineurin inhibitor

^a^ Steady-state dose-adjusted concentration (C/dose)

### Gene allele frequencies

Genotype and allele frequencies of the CYP3A5*3 and CYP3A4*22 variants are presented in Table 2. We observed no significant linkage disequilibrium between CYP3A4*22 and CYP3A5*3 alleles (*P* = 0.69). We observed significant linkage disequilibrium between the two *PPARA* sequence variants (*P* < 0.001). Owing to this significant correlation, these genotypes were combined into a new ad hoc variable for further analysis; *PPARA* variant allele carriers (one or two variant alleles of either *PPARA* c.209-1003G>A or *PPARA* c.208 + 3819A>G, *n* = 60) and *PPARA* wild-types (*n* = 63). None of the genotype frequencies deviated from the Hardy–Weinberg distribution, *P* > 0.7, Chi-squared test (Table 2).

**Table 2:** Genotype and allele frequencies in the study population (*n* = 177) compared with allele frequencies reported in the literature

	Genotype frequencies, *n* (%)			Allele frequencies (%)	Reported allele frequencies in Caucasians^b^ (%)
	AA	Aa	aa		
CYP3A5*3 ^a^	142 (80)	34 (19)	1 (1)	90	81–96
CYP3A4*22	168 (95)	9 (5)	0 (0)	3	3–6^c^
*PPARA* c.209-1003G>A	108 (61)	59 (33)	10 (6)	22	21–24
*PPARA* c.208 + 3819A>G	100 (56)	63 (36)	14 (8)	26	25–26
POR*28	82 (46)	78 (44)	17 (10)	32	28–31

*AA* homozygote carriers of the major allele, *Aa* heterozygote carriers, *aa* homozygote carriers of the minor (variant) allele

^a^Variant allele is predominant in Caucasians

^b^ http://www.ncbi.nlm.nih.gov/variation/tools/1000genomes/

^c^Allele frequency based on published studies [14, 17–19, 31, 32]

#### Impact of genotypes on tacrolimus C_0_/D ratio

**Fig. 1:**
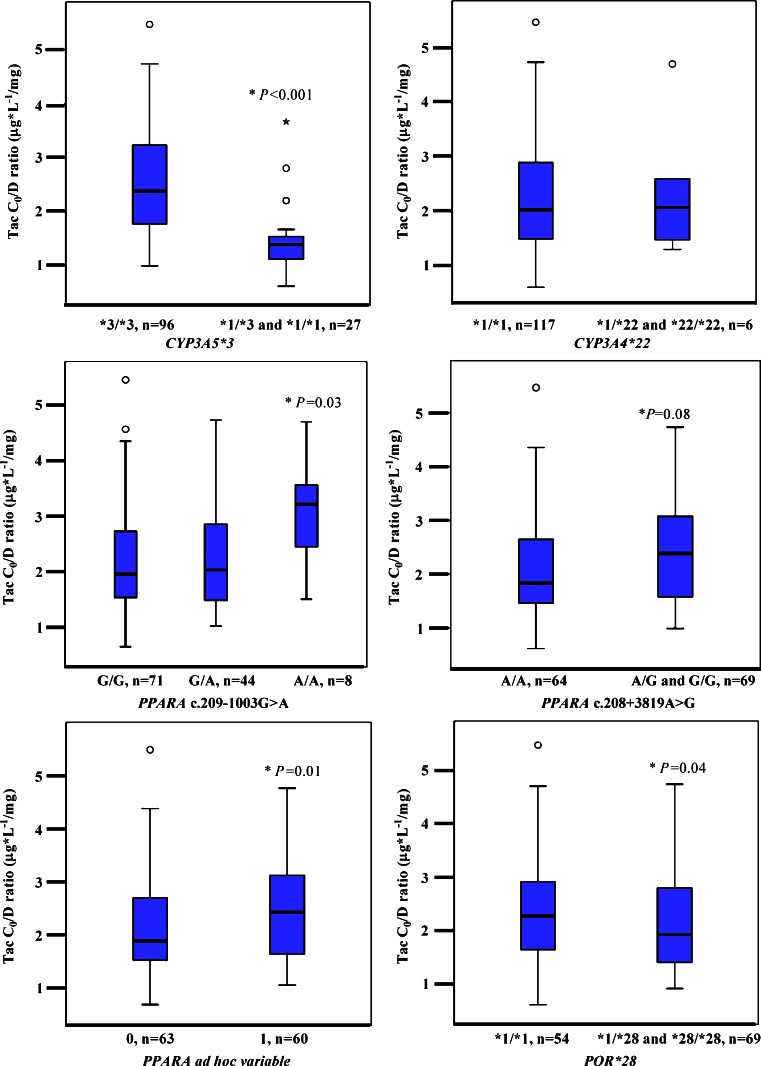
Tacrolimus C_0_/D ratio (μg*L^-1^/mg) as a function of CYP3A5*3, CYP3A4*22, PPARA c.209-1003G>A*, PPARA c.208 + 3819A>G*, and POR*28. The box-and-whisker plots indicate interquartile ranges (*boxes*), medians (*horizontal lines in the boxes*), and the highest and lowest values (*whiskers above and below the boxes*). *P* values are related to the ANOVA test, described under Results. C_0_/D, dose-adjusted concentrations before dosing; *CYP,* gene encoding cytochrome P450; *PPARA,* gene encoding the nuclear receptor peroxisome proliferator-activated receptor alpha; *POR,* gene encoding cytochrome P450 oxidoreductase

Heterozygous CYP3A5*1 recipients showed 42 % lower mean C_0_/D ratio (1.38 ± 1.07 μg*L^-1^/mg) compared with homozygote carriers of CYP3A5*3 (2.34 ± 1.04 μg*L^-1^/mg; *P* < 0.001; Fig. 1). A multivariate analysis accounting for the other genotypes investigated showed that the CYP3A5*1 allele was an independent explanatory factor for the Tac C_0_/D ratio. A correlation analysis revealed that the CYP3A5*1 genotype explained approximately 25 % of the interindividual variability in Tac dose-adjusted trough concentration (r^2^ = 0.249, *n* = 177, *P <* 0.001). No association was found between the CYP3A4*22 and Tac C_0_/D ratio (Fig. 1, Table 3). Application of the *CYP3A* genotype-based classification system published by Elens et al. with combined *CYP3A* allelic status did not give any additional information in this study (data not shown) [19].

**Table 3:** Tacrolimus and cyclosporine median C/D ratios in different genotypes

	Tac C_0_/D ratio (μg*L^-1^/mg)	*n*	CsA C_2_/D ratio (μg*L^-1^/mg)	*n*
CYP3A5*3				
*1/*1	-	0	6.89	1
*1/*3	1.38 (0.61, 3.67)	27	7.62 (6.53, 10.93)	7
*3/*3	2.37 (0.98, 5.47)	96	7.16 (2.24, 16.20)	46
CYP3A4*22				
*1/*1	2.03 (0.61, 5.47)	117	7.11 (2.24, 16.20)	51
*1/*22	2.07 (1.30, 4.70)	6	11.12 (8.70, 11.17)	3
*22/*22	-	0	-	0
*PPARA* c.209-1003G>A				
GG	1.93 (0.61, 5.47)	71	7.06 (2.46, 16.20)	37
GA	2.01 (0.99, 4.73)	44	7.60 (2.24, 11.17)	15
AA	3.20 (1.47, 4.70)	8	6.36 (5.48, 7.25)	2
*PPARA* c.208 + 3819A>G				
AA	1.83 (0.61, 5.47)	64	7.09 (2.46, 16.20)	36
AG	2.27 (0.99, 4.73)	47	7.58 (2.24, 11.17)	16
GG	2.70 (1.30, 4.70)	12	6.36 (5.48, 7.25)	2
POR*28				
*1/*1	2.27 (0.61, 5.47)	54	7.24 (2.24, 11.12)	28
*1/*28	1.88 (0.92, 4.73)	62	6.82 (3.54, 16.20)	16
*28/*28	1.95 (1.11, 3.40)	7	7.98 (4.87, 11.17)	10

Data are presented as median (range) unless otherwise stated

The Tac C_0_/D ratio was 15 % higher (*P* = 0.08) in *PPARA* variant allele carriers (*PPARA* ad hoc variable), 2.24 ± 1.06 μg*L^-1^/mg compared with 1.95 ± 1.05 μg*L^-1^/mg in *PPARA* wild types. After adjusting for the other genotypes (POR*28, CYP3A5*3, and CYP3A4*22), Tac dose-adjusted trough concentrations were significantly higher among *PPARA* variant allele carriers; 1.93 ± 1.09 μg*L^-1^/mg vs 1.63 ± 1.09 μg*L^-1^/mg in wild-type carriers (19 %, *P* = 0.01). However, individual analyses of each *PPARA* variant allele indicated some differences in the effect of the two variants. A one-way analysis of variance with *PPARA* c.208 + 3819A>G as the only independent variable revealed a non-significant 15 % higher C_0_/D ratio for *PPARA* c.208 + 3819 G variant (2.24 ± 1.06 μg*L^-1^/mg) vs wild-type allele carriers (1.95 ± 1.05 μg*L^-1^/mg), *P* = 0.08. When additionally accounting for CYP3A5*3, CYP3A4*22, *PPARA* c.209-1003G>A, and POR*28 genotypes, the C_0_/D ratio was, however, significantly higher (35 %, *P* = 0.02) in patients carrying at least one *PPARA* c.208 + 3819 G allele (AG/GG 2.06 ± 1.11 μg*L^-1^/mg vs AA 1.52 ± 1.11 μg*L^-1^/mg), indicating that *PPARA* c.208 + 3819A>G has an independent impact on Tac C_0_/D ratio (Fig. 1).

There was a 44 % higher Tac C_0_/D ratio for homozygote *PPARA* c.209-1003AA carriers (2.92 ± 1.16 μg*L^-1^/mg) compared with homozygote *PPARA* c.209-1003GG carriers (2.02 ± 1.05 μg*L^-1^/mg; *P* = 0.03; Fig. 1, Table 3). Including CYP3A5*3, CYP3A4*22, *PPARA* c.208 + 3819A>G, and POR*28 as fixed factors in the analysis of variance, the impact of *PPARA* c.209-1003G >A on Tac trough concentrations was reduced. No significant difference was detected between *PPARA* c.209-1003GG carriers and heterozygote carriers of the variant allele or the group of homo- and heterozygote carriers of the *PPARA* c.209-1003A allele and *PPARA* c.209-1003GG carriers.

Carriers of the POR*28 variant allele carriers tended to have a lower Tac C_0_/D ratio (10 %) 1.99 ± 1.05 μg*L^-1^/mg compared with 2.21 ± 1.06 μg*L^-1^/mg in POR*28 wild type (*P* = 0.19). After including CYP3A5*3, CYP3A4*22, and *PPARA* genotype as fixed factors, homozygous and heterozygous POR*28 carriers demonstrated significantly lower C_0_/D ratio (1.65 ± 1.09 μg*L^-1^/mg) compared with patients homozygous for the *POR* wild-type allele (1.90 ± 1.09 μg*L^-1^/mg; 15 %, *P* = 0.04; Fig. 1).

#### Impact of genotypes on cyclosporine C_2_/D ratio

**Fig. 2:**
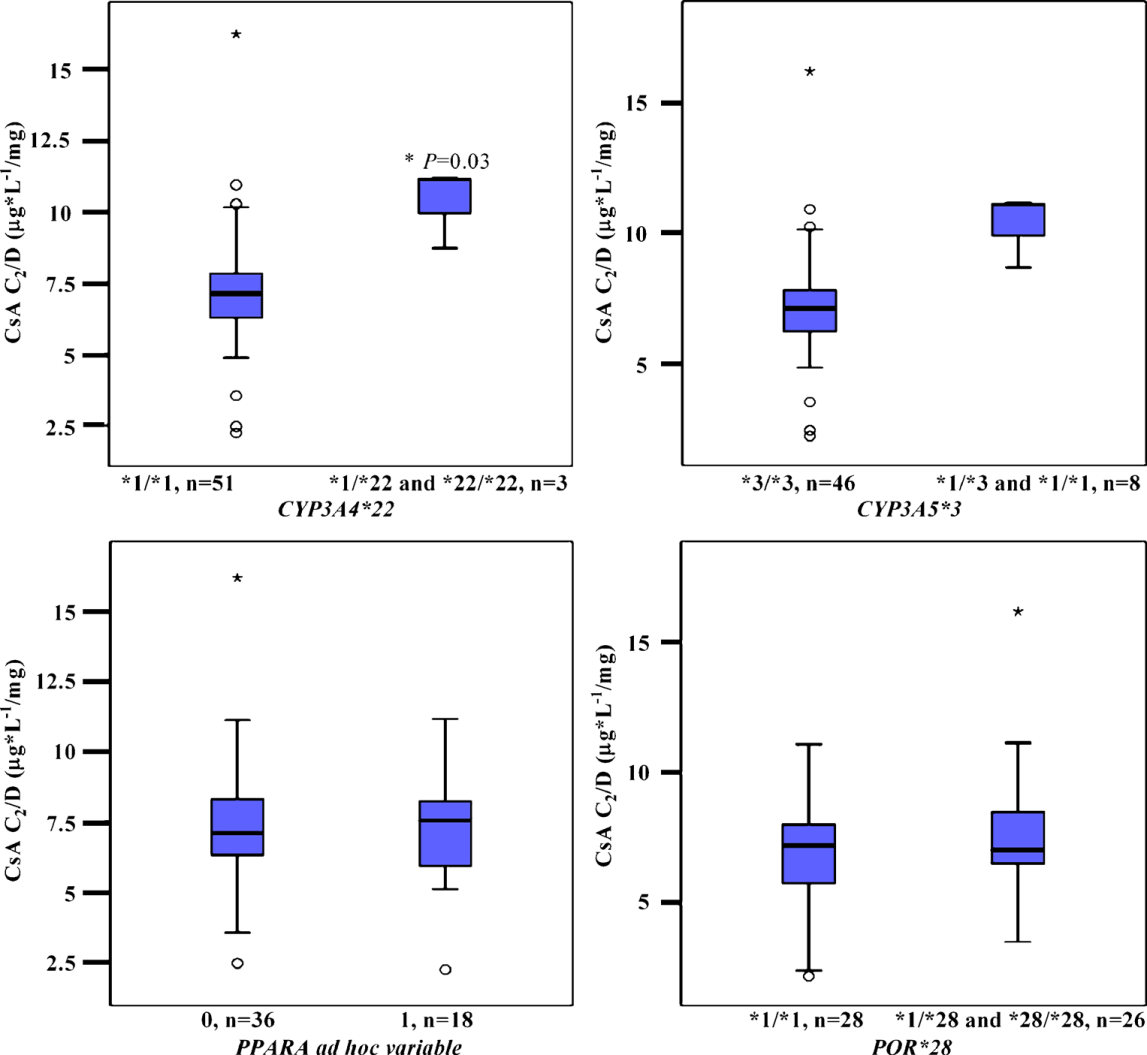
Cyclosporine C_2_/D ratio (μg*L^-1^/mg) as a function of CYP3A5*3, CYP3A4*22, PPARA c.209-1003G>A*, PPARA* c.208 + 3819A>G, and POR*28. The box-and-whisker plots indicate interquartile ranges (*boxes*), medians (*horizontal lines in the boxes*), and highest and lowest values (*whiskers above and below the boxes*). *P* values are related to the ANOVA test, as described under Results. C_2_/D, dose-adjusted concentrations before dosing; *CYP,* gene encoding cytochrome P450; *PPARA,* gene encoding the nuclear receptor peroxisome proliferator-activated receptor alpha; *POR,* gene encoding cytochrome P450 oxidoreductase

In the CsA group the CYP3A5*3 genotype did not show any statistical significant influence on C_2_/D ratio (*P* = 0.31; Fig. 2). However, renal transplant recipients carrying the CYP3A4*22 allele showed significantly higher dose-adjusted CsA C_2_ levels. Univariate analysis revealed that CYP3A4*1/*22 carriers demonstrated 50 % higher mean C_2_/D ratios (10.26 ± 1.20 μg*L^-1^/mg) compared with homozygote CYP3A4*1/*1 carriers (6.84 ± 1.04 μg*L^-1^/mg; *P* = 0.04; Fig. 2, Table 3). Adjusting for the other genotypes in a multivariate analysis confirmed an independent effect of CYP3A4*22 on CsA pharmacokinetics, with a 53 % higher CsA C_2_/D ratio (10.91 ± 1.22 μg*L^-1^/mg) among variant allele carriers compared with wild-type carriers (7.12 ± 1.07 μg*L^-1^/mg), *P* = 0.03.The CYP3A4*22 allele explained approximately 12 % of the interindividual variability in the CsA C_2_/D ratio (r = 0.35, *P <* 0.01).

No statistical significant associations were observed between *PPARA* (*P* = 0.85, *P* = 0.74) or POR*28 (*P* = 0.27, *P* = 0.52) sequence variants and the CsA C_2_/D ratio.

## Discussion

The present study demonstrated that Tac and CsA pharmacokinetics are influenced by sequence variants in several genes. We confirmed the previously well-described effect of CYP3A5*1 on Tac exposure, but did not find any association between CYP3A5*1 and CsA exposure. *PPARA* variant alleles and the POR*28 allele were associated with higher and lower Tac C_0_/D ratios respectively, while the CYP3A4*22 allele influenced CsA C_2_/D ratios.

Carriers of functional CYP3A5*1 alleles demonstrated a 58 % lower Tac C_0_/D ratio (*P* < 0.001). This confirms the significance of the *CYP3A5* genetic polymorphism on Tac metabolism previously shown in several publications, where carriers of CYP3A5*1 alleles (CYP3A5 expressers) have been reported to require about twice the doses of Tac compared with CYP3A5*3/*3 individuals [9, 10, 29]. Thus, pre-transplant *CYP3A5* genotyping may be a useful approach for better prediction of individual Tac starting doses.

Based on the CsA C_2_ data, the present study also supports the significant impact of the newly identified CYP3A4*22 allele on the metabolism of CYP3A substrates [14]. Even though the effect of knowing this genotype in a Caucasian population is limited, the individual influence in those few carrying this variant allele is substantial. A rough estimate is that recipients with one or two CYP3A4*22 alleles need half the dose of CsA to reach the therapeutic target. Somewhat surprisingly, we did not observe any association between CYP3A4*22 genotype and Tac C_0_/D ratios. This observed differential effect of the CYP3A4*22 genotype on Tac and CsA pharmacokinetics, may be due to a difference in preferred metabolic pathways, CYP3A4 vs CYP3A5, for the two drugs. Additionally, there were no recipients homozygous for the CYP3A4*22 variant allele among the 123 patients treated with Tac, which may have confounded the results. In contrast to the present findings, Elens et al. reported a significant association between CYP3A4*22 and both Tac and CsA pharmacokinetics, reporting higher dose-adjusted CsA and Tac concentrations in kidney transplant recipients [15, 18, 19]. However, these authors failed to confirm the association between CsA C/D ratio and CYP3A4*22 in an independent cohort [30]. Although there seems to be an international agreement on the reduced CYP3A4 metabolic capacity among CYP3A4*22 carriers, further investigations are required to clarify the clinical relevance of this sequence variant in patients treated with Tac and CsA.

To our knowledge, this is the first study showing the potential impact of the *PPARA* genetic variations on Tac exposure in kidney transplant recipients. Owing to the strong correlation between *PPARA* c.209-1003G>A and *PPARA* c.208 + 3819A>G, the combined effect of these two sequence variants was analyzed. The independent effect of expressing at least one *PPARA* variant allele was significantly associated with a higher Tac C_0_/D ratio (*P* = 0.01), when adjusting for the other sequence variants (POR*28, CYP3A5*3, and CYP3A4*22). A detailed analysis of the two *PPARA* sequence variants showed significantly increased Tac exposure in homozygote *PPARA* c.209-1003G>A carriers. These results are in concordance with the reduced CYP3A4 protein/activity levels previously presented [16]. However, inclusion of the other sequence variants assessed in the present study reduced the effect of *PPARA* c.209-1003G>A on Tac C_0_/D ratios, indicating other possible explanatory variables in addition to the difference observed in Tac C_0_/D ratios between homozygote *PPARA* c.209-1003G>A carriers and homozygote *PPARA* c.209-1003G carriers. On the other hand, expression of at least one *PPARA* c.208 + 3819G allele was an independent explanatory factor for higher Tac exposure. This suggests that *PPARA* c.208 + 3819A>G is the *PPARA* sequence variant with the strongest influence on Tac pharmacokinetics.

Despite the statistically significant effect of *PPARA* sequence variants on Tac exposure, no significant effect was shown on CsA C_2_/D ratios. Although the mechanism is not fully understood, activation of PPAR-alpha has been shown to increase expression of CYP3A4. Consequently, PPAR-alpha activity should theoretically also have influenced CsA pharmacokinetics [16]. However, there are inconsistent reports on whether the regulation of CYP3A4 occurs directly or indirectly by PPAR-alpha [16, 31, 32]. Recently, the sequence variants *PPARA* c.209-1003G>A and *PPARA* c.208 + 3819A>G were associated with reduced expression of PPAR-alpha, and consistently related to lower *CYP3A4* mRNA levels, protein expression, and enzymatic activity [16, 33]. PPAR-alpha has been linked to CYP3A4 expression, but an association between PPAR-alpha and CYP3A5 expression and activity has not yet been reported. The association between *PPARA* gene variants and Tac, but not CsA, pharmacokinetics may be at least partly related to different metabolic pathways and different regulation mechanisms of CYP3A4 and CYP3A5 expression and activity.

The significantly lower Tac C_0_/D ratio observed among POR*28 allele carriers after correction for CYP3A5*3, CYP3A4*22 and *PPARA* genotype supports the previous findings of De Jonge et al. and Oneda et al. [24, 25], reporting a lower Tac C_0_/D ratio among POR*28 allele carriers expressing functional CYP3A5. However, the present study did not show any significant impact of the POR*28 allele on Tac C_0_/D ratio in the sub-group of patients expressing functional CYP3A5. The POR*28 allele has the potential to explain interindividual variability in CYP3A capacity. However, the proposed link between CYP3A5 and the POR*28 allele needs further elucidation.

### Study limitations

The sample size of the CsA subgroup may limit some aspects of the present study, and low CYP3A4*22 and CYP3A5*1 allele frequencies may also explain part of the discrepancy between observations in the present study and the literature. Only one recipient homozygous for CYP3A5*1 were detected in this study, and we therefore cannot exclude a potential effect of CYP3A5*1 status on POR*28, nor can we exclude an association between CYP3A4*22 and Tac pharmacokinetics. The rarity of the minor homozygous allele populations of all the SNVs tested, in addition to multiple testing, will have a significant impact on the power of the study.

### Clinical relevance

The results suggest an impact of the two linked *PPARA* sequence variants and POR*28, in addition to CYP3A5*3, on Tac pharmacokinetics, as well as an influence of CYP3A4*22 on CsA pharmacokinetics. Genotyping pre-transplant may allow better individualization of initial CNI doses and thereby reduce the risk of CNI over- and under-exposure in the critical phase immediately after transplantation. Determination of a combination of relevant gene variants seems to allow even more optimal dosage predictions than *CYP3A5* genotyping alone. However, because of the relatively small effect size of the two SNV in *PPARA* and POR*28, the clinical applicability of the genetic testing of these sequence variants needs to be further investigated in even larger cohorts.

## Conclusion

In conclusion, we confirmed that CYP3A5*1 is significantly associated with lower Tac C_0_/D ratio in kidney transplant recipients. Further, our results suggest that POR*28 and *PPARA* variant alleles (c.209-1003G>A and c.208 + 3819A>G), in addition to CYP3A5*3, might influence Tac exposure, and that CYP3A4*22 status is of importance for CsA pharmacokinetics. Interestingly, the POR*28 allele influenced Tac exposure independent of the CYP3A5*3 status, contrary to what has previously been hypothesized, and the CYP3A4*22 allele was identified as a significant independent predictor of CsA exposure. Pre-transplant genotyping of these sequence variants may help to identify renal transplant recipients at risk of CNI over- or underexposure, and contribute to reducing CNI-related adverse events by more optimal determination of individual starting doses.

## Electronic supplementary material

Below is the link to the electronic supplementary material.

ESM 1 (DOCX 15 kb)

ESM 2 (DOCX 16 kb)

ESM 3 (DOCX 15 kb)

ESM 4 (DOCX 16 kb)
