# Supplementary material for: The influence of CYP3A, PPARA, and POR genetic variants on the pharmacokinetics of tacrolimus and cyclosporine in renal transplant recipients
Source: Eur J Clin Pharmacol. 2014 Mar 22;70(6):685–93. doi: 10.1007/s00228-014-1656-3 (PMC4025175; doi:10.1007/s00228-014-1656-3)
Supplement: Supplementary file 1 — (DOCX 15 kb) [file 228_2014_1656_MOESM1_ESM.docx]

**The influence of *CYP3A*, *PPARA* and *POR* genetic variants on the pharmacokinetics of tacrolimus and cyclosporine in renal transplant recipients**

**European Journal of Clinical Pharmacology**

Ingrid Lunde^1^, Sara Bremer^2^, Karsten Midtvedt^3^, Beata Mohebi^1^, Miriam Dahl^1^, Stein Bergan^1,4^, Anders Åsberg^1,3^ and Hege Christensen^1^

^1^Department of Pharmaceutical Biosciences, School of Pharmacy, University of Oslo

^2^Department of Medical Biochemistry, Oslo University Hospital, Rikshospitalet

^3^Laboratory for Renal Physiology, Medical Department, Oslo University Hospital, Rikshospitalet

^4^Department of Pharmacology, Oslo University Hospital, Rikshospitalet

Correspondence: Ingrid Lunde, School of Pharmacy, University of Oslo, Box 1068 Blindern, N-0316 Oslo, Norway. Telephone: +47 22 85 75 19, Fax: + 47 22 85 44 02. E-mail: Ingrid.lunde@farmasi.uio.no.

**Online resource table 1**. Primer sequences and restriction enzymes for *POR* and *PPARA* genotyping

| Assay | Sequence (5´ to 3´) | Product length | Restriction enzyme |
| --- | --- | --- | --- |
| *POR*28*  Forward primer  Reverse primer | CATCTGTGCGGTGGTTGT  TGAAGGGCAGGCGGA | 158 bp | FastDigest® MspI |
| *PPARA* (c.209-1003G>A)  Forward primer  Reverse primer | TCTCCCAGTCTGTGGCTTGT  ATCTCCGGACCCACACATC | 372 bp | FastDigest® EcoRI |
| *PPARA* (c.208+3819A>G)  Forward primer  Reverse primer | CTGACAGAGGTAAGGCTT  ATTTAGATGGGAAGCACA | 104 bp | FastDigest® Tsp509I |
