# Supplementary material for: The influence of CYP3A, PPARA, and POR genetic variants on the pharmacokinetics of tacrolimus and cyclosporine in renal transplant recipients
Source: Eur J Clin Pharmacol. 2014 Mar 22;70(6):685–93. doi: 10.1007/s00228-014-1656-3 (PMC4025175; doi:10.1007/s00228-014-1656-3)
Supplement: Supplementary file 2 — (DOCX 16 kb) [file 228_2014_1656_MOESM2_ESM.docx]

**The influence of *CYP3A*, *PPARA* and *POR* genetic variants on the pharmacokinetics of tacrolimus and cyclosporine in renal transplant recipients**

**European Journal of Clinical Pharmacology**

Ingrid Lunde^1^, Sara Bremer^2^, Karsten Midtvedt^3^, Beata Mohebi^1^, Miriam Dahl^1^, Stein Bergan^1,4^, Anders Åsberg^1,3^ and Hege Christensen^1^

^1^Department of Pharmaceutical Biosciences, School of Pharmacy, University of Oslo

^2^Department of Medical Biochemistry, Oslo University Hospital, Rikshospitalet

^3^Laboratory for Renal Physiology, Medical Department, Oslo University Hospital, Rikshospitalet

^4^Department of Pharmacology, Oslo University Hospital, Rikshospitalet

Correspondence: Ingrid Lunde, School of Pharmacy, University of Oslo, Box 1068 Blindern, N-0316 Oslo, Norway. Telephone: +47 22 85 75 19, Fax: + 47 22 85 44 02. E-mail: Ingrid.lunde@farmasi.uio.no.

**Online resource table 2**. Amplification conditions

|  | **Denaturation** | **Amplification** | | |  |
| --- | --- | --- | --- | --- | --- |
|  |  | **Denaturation** | **Annealing** | **Elongation** | **Cycles** |
| *CYP3A5*3* | 95°C for 5 min | 95°C for 10 sec | 63°C for 10 sec | 72°C for 30 sec | 40 |
| *CYP3A4*22* | 95°C for 5 min | 95°C for 10 sec | 63°C for 10 sec | 72°C for 30 sec | 40 |
| *PPARA* c.209-1003G>A | 94°C for 5 min | 94°C for 30 sec | 62°C for 20 sec | 72°C for 30 sec | 40 |
| *PPARA* c.208+3819A>G | 94°C for 5 min | 94°C for 30 sec | 60°C for 20 sec | 72°C for 30 sec | 40 |
| *POR*28* | 94°C for 5 min | 94°C for 30 sec | 62°C for 20 sec | 72°C for 30 sec | 40 |
