# Supplementary material for: The influence of CYP3A, PPARA, and POR genetic variants on the pharmacokinetics of tacrolimus and cyclosporine in renal transplant recipients
Source: Eur J Clin Pharmacol. 2014 Mar 22;70(6):685–93. doi: 10.1007/s00228-014-1656-3 (PMC4025175; doi:10.1007/s00228-014-1656-3)
Supplement: Supplementary file 3 — (DOCX 15 kb) [file 228_2014_1656_MOESM3_ESM.docx]

**The influence of *CYP3A*, *PPARA* and *POR* genetic variants on the pharmacokinetics of tacrolimus and cyclosporine in renal transplant recipients**

**European Journal of Clinical Pharmacology**

Ingrid Lunde^1^, Sara Bremer^2^, Karsten Midtvedt^3^, Beata Mohebi^1^, Miriam Dahl^1^, Stein Bergan^1,4^, Anders Åsberg^1,3^ and Hege Christensen^1^

^1^Department of Pharmaceutical Biosciences, School of Pharmacy, University of Oslo

^2^Department of Medical Biochemistry, Oslo University Hospital, Rikshospitalet

^3^Laboratory for Renal Physiology, Medical Department, Oslo University Hospital, Rikshospitalet

^4^Department of Pharmacology, Oslo University Hospital, Rikshospitalet

Correspondence: Ingrid Lunde, School of Pharmacy, University of Oslo, Box 1068 Blindern, N-0316 Oslo, Norway. Telephone: +47 22 85 75 19, Fax: + 47 22 85 44 02. E-mail: Ingrid.lunde@farmasi.uio.no.

**Online resource table 3.** Oligonucleotide sequences

| **Gene** | **Name** | **Oligonucleotide** | **Sequence (5´-3´)** |
| --- | --- | --- | --- |
|  |  |  |  |
| *CYP3A4*22* |  |  |  |
|  | CYP3A4-22_F1 | Forward primer | AGCCCCTTAGGAAGAGTT |
|  | CYP3A4-22_R1 | Reverse primer | AGAAGGTGTTATCAGGTGC |
|  | CYP3A4-22_FL1 | Donor probe | CTTGATCTCAGAGGTAGGTCTAATTCAGTTCAGTG-Fluorescein |
|  | CYP3A4-22_LC1 | Acceptor probe | LC Red 640-TCCATCACACCCAGCGTAGG-Phosphate |
|  |  |  |  |
| *CYP3A5*3* |  |  |  |
|  | CYP3A5-3_F5 | Forward primer | ATGGAGAGTGGCATAGGA |
|  | CYP3A5-3_R4 | Reverse primer | ACACACAGCAAGAGTCTCA |
|  | CYP3A5-3_LC3 | Donor probe | GAGCTCTTTTGTCTTTCAATATCTCT-Fluorescein |
|  | CYP3A5-3_LC4 | Acceptor probe | LC640- CCCTGTTTGGACCACATTACCCTT -Fosfat |
