# Supplementary material for: The influence of CYP3A, PPARA, and POR genetic variants on the pharmacokinetics of tacrolimus and cyclosporine in renal transplant recipients
Source: Eur J Clin Pharmacol. 2014 Mar 22;70(6):685–93. doi: 10.1007/s00228-014-1656-3 (PMC4025175; doi:10.1007/s00228-014-1656-3)
Supplement: Supplementary file 4 — (DOCX 16 kb) [file 228_2014_1656_MOESM4_ESM.docx]

**The influence of *CYP3A*, *PPARA* and *POR* genetic variants on the pharmacokinetics of tacrolimus and cyclosporine in renal transplant recipients**

**European Journal of Clinical Pharmacology**

Ingrid Lunde^1^, Sara Bremer^2^, Karsten Midtvedt^3^, Beata Mohebi^1^, Miriam Dahl^1^, Stein Bergan^1,4^, Anders Åsberg^1,3^ and Hege Christensen^1^

^1^Department of Pharmaceutical Biosciences, School of Pharmacy, University of Oslo

^2^Department of Medical Biochemistry, Oslo University Hospital, Rikshospitalet

^3^Laboratory for Renal Physiology, Medical Department, Oslo University Hospital, Rikshospitalet

^4^Department of Pharmacology, Oslo University Hospital, Rikshospitalet

Correspondence: Ingrid Lunde, School of Pharmacy, University of Oslo, Box 1068 Blindern, N-0316 Oslo, Norway. Telephone: +47 22 85 75 19, Fax: + 47 22 85 44 02. E-mail: Ingrid.lunde@farmasi.uio.no.

**Online resource table 4**. Reaction mixtures for the amplification of target genes

|  | **Target genes** | | | | |
| --- | --- | --- | --- | --- | --- |
|  | ***CYP3A5*** | ***CYP3A4*** | ***PPARA***  (***c.209-1003G>A)*** | ***PPARA*** (***c.208+3819A>G)*** | ***POR*** |
| Forward primer (µmol/L) | 0.05 | 0.25 | 0.8 | 0.8 | 0.4 |
| Reverse primer (µmol/L) | 0.15 | 0.75 | 0.4 | 0.8 | 0.8 |
| Donor probe (µmol/L) | 0.15 | 0.15 | - | - | - |
| Acceptor probe (µmol/L) | 0.15 | 0.15 | - | - | - |
| Deoxynucleotide (dNTP) mix (mmol/L) | - | - | 0.2 | 0.2 | 0.2 |
| MgCl_2_ (mmol/L) | - | - | 2.0 | 1.5 | 1.0 |
| Taq DNA polymerase (U/µL) | - | - | 0.025 | 0.025 | 0.025 |
| H_2_O, PCR-grade (µL) | q.s. | q.s. | q.s. | q.s. | q.s. |
| LightCycler Probes Master 2x (µL) | 10 | 10 | - | - | - |
| DNA (µL) | 5 | 5 | 4 | 4 | 4 |
|  |  |  |  |  |  |
| Final volume (µL) | 20 | 20 | 50 | 50 | 50 |
